# Application of Growth Hormone in *in vitro* Fertilization

**DOI:** 10.3389/fendo.2019.00502

**Published:** 2019-07-23

**Authors:** Yue-Ming Xu, Gui-Min Hao, Bu-Lang Gao

**Affiliations:** ^1^Department of Reproductive Medicine, The Second Hospital, Hebei Medical University, Shijiazhuang, China; ^2^Department of Medical Research, Shijiazhuang First Hospital, Hebei Medical University, Shijiazhuang, China

**Keywords:** growth hormone, *in vitro* fertilization, infertility, reproduction, effect

## Abstract

Growth hormone (GH) is a peptide hormone secreted mainly by the anterior part of the pituitary gland and plays a critical role in cell growth, development, and metabolism throughout the body. GH can not only directly influence human oocytes and cumulus cells but also indirectly improve oocyte quality through activating synthesis of insulin-like growth factor-I or promoting follicle-stimulating hormone-induced ovarian steroidogenesis. Since GH can regulate female and male infertility, it has been applied in the management of infertility for many years, especially in patients with poor ovarian response or poor prognosis. During ovarian stimulation, GH administration might improve the success rate of *in vitro* fertilization (IVF) probably through the beneficial effects of GH on oocyte quality as indicated by a higher number of mature oocytes and embryos arriving at the transfer stage and a higher fertility rate in GH-treated patients. However, there is still great controversy in the application of GH in IVF. While some researchers showed that pregnancy, implantation and live birth rates could be increased by ovarian pretreatment with GH, others did not support GH as an effective adjuvant for infertility treatment because the live birth rate was not increased. This study reviewed and summarized recent advancements and benefits in clinical application of GH, trying to reach a just unbiased conclusion regarding the effect of GH therapy in IVF.

## Introduction

As a peptide hormone secreted mainly by the anterior part of the pituitary gland in a pulsatile manner, growth hormone (GH) plays a critical role in cell growth, development and metabolism throughout the body with multifunctional effects ranging far beyond the effect on linear growth (1). Human oocytes and cumulus cells have GH receptors (GHRs) and can be directly influenced by GH, and GH can promote nuclear maturation of denuded human oocytes (2–4). GH may also have an indirect effect on improving oocyte quality through activating synthesis of insulin-like growth factor (IGF)-I or promoting follicle-stimulating-hormone-induced ovarian steroidogenesis (5, 6). Since GH is involved in the regulation of female and male infertility, it has consequently been applied in the management of infertility for many years (7), especially in patients with poor ovarian response or poor prognosis (8–11). During ovarian stimulation, GH administration can improve the success rate of *in vitro* fertilization (IVF) (12, 13) probably through the beneficial effects of GH on oocyte quality as indicated by a higher number of mature oocytes and embryos arriving at the transfer stage and a higher fertility rate in GH-treated patients (8, 10, 11, 14). Pregnancy, implantation and live birth rates can be increased by ovarian pretreatment with GH in many studies (5, 9, 12, 13, 15–17), and subgroup analysis in a systematic review and meta-analysis indicated that GH administration with gonadotropin significantly increased the clinical pregnancy (risk ratio (RR) 1.76, 1.25–2.48), live birth rate (RR 1.91, 1.29–2.83), collected oocytes number (standard mean difference (SMD) 1.09, 95% CI 0.54–1.64), MII oocytes number (SMD 1.48, 95% CI 0.84–2.13) and E2 on human chorionic gonadotropin day (SMD 1.03, 95% CI 0.18–1.89) among patients with poor ovarian responses (11). However, great controversy still exists in the application of GH in IVF, with some randomized controlled trials presenting no definitive benefits in increasing the live birth rate for poor responders (18, 19). Dakhly et al. reported non-significant (*P* > 0.05) improvement in the live birth (17.5 vs. 14.1%) and cumulative live birth rate (18.3 vs. 14.7%) in a randomized controlled trial (*n* = 120 patients for each group) with addition of GH as an adjuvant therapy [2.5 mg or 7.5 IU GH injected subcutaneously from day 21 of the previous cycle along with GnRHa until the day of human chorionic gonadotropin (hCG)] to the long down regulation protocol in poor responders undergoing IVF, even though GH significantly (*P* < 0.001) increased the endometrial thickness (11.8 ± 1.3 vs. 11.3 ± 1.2 mm), number of collected oocytes (5.4 ± 1.7 vs. 4.3 ± 2.1), MII oocytes (4.1 ± 2.1 vs. 2.1 ± 1.4), fertilized oocytes (4.0 ± 2.2 vs. 2.0 ± 1.2), transferred embryos (2.4 ± 0.9 vs. 1.6 ± 1.1) and frozen embryos (1.1 ± 1.4 vs. 0.2 ± 0.5) (18). However, their outcomes were questioned by Yovich et al. (20) who believed that this randomized controlled trial suffered from some major limitations (including suboptimal dosage of Cyclogest 400 mg twice daily in the luteal phase management and use of a non-single embryo transfer strategy) which strongly weakened the effects. Dakhly et al. also acknowledged that further studies were needed to investigate the true impact of adding GH to the induction protocols in poor responders (20). In the most recent randomized placebo controlled trial investigating the effect of adding human GH to an IVF cycle in previously documented poor responders to FSH with GH administered at the dose of 12 IU starting on day 1 of stimulation and stopped on the evening of hCG scheduling for egg pickup (19), a significant increase in the live birth rate (14.5% for GH vs. 13.7% for placebo, with an odds ratio (OR) 1.07 and 95% CI between 0.37 and 3.10) from the addition of GH could not be found. However, this clinical trial suffers from too many limitations and has to be interpreted with caution. To achieve the goal of increasing the live birth rate from 10 to 20% by addition of GH according to previous systematic reviews and ability to recruit, 195 participants were needed for each arm in order to reach the 5% significant level, 80% statistical power and 10% dropout or cancellation rate, however, only 65 patients were recruited and assigned to either the GH group or the controlled placebo group from four clinical centers. This indicated that the trial was not a successfully completed trial without sufficient statistical power, implying that their outcomes were underpowered. This clinical trial lasted 4 years and was ended early as the provided drug had expired, indicating that this trial was terminated because of the expired drug rather than insufficient effect of GH administration on these poor-responding patients. Usually, when a clinical trial is terminated ahead of time, it is because the targeted endpoint has been proven in advance or because of substantial side effects of the target drug. However, this trial was terminated in advance because of the expired drug without even achieving the planned endpoints or goal of study. Moreover, as stated by the authors, some women who had good responses were also enrolled because different definition of poor responders was adopted (their definition does not fit with all classical international criteria) in this trial, to say nothing of many other limitations listed at the end of the study (19). Since different opinions exist in the application and benefit of GH adjuvant therapy in IVF, the present review aims to review and summarize recent advancements and benefits in clinical application of GH, trying to reach a just unbiased conclusion regarding the effect of GH adjuvant therapy in IVF based on the following aspects: theoretic bases of GH application, GH application in IVF, administration protocol and subjects and benefits of GH application (Table 1).

**Table 1:** Effects and application of growth hormone in *in vitro* fertilization.

**Theoretic bases of GH application**	**GH application in IVF**	**Administration protocol**	**Subjects and benefits of GH**
Signaling pathway	Improving ovarian response	4–6 w before hCG administration	Subjects: poor or normal ovarian responders
On ovarian reactivity	Improving oocyte quality	Luteal phase of preceding menstrual cycle	Subjects: poor quality of embryos
On follicle development	Improving uterine receptivity	Start of gonadotropin	Subjects: improper endometrial reaction
On endometrial receptivity		Middle and late follicular phases	Subjects: repeated implantation failure
			Benefit: increased live birth rate

*GH, growth hormone; IVF, in vitro fertilization; hCG, human chorionic gonadotropin*.

## Theoretical Bases for GH Application in IVF

GH is a key factor for optimal fertility in women, which has been proved by declined fertility in GH deficiency women and capability of GH replacement to capacitate successful unassisted pregnancies in previously infertile women with GH deficiency (21, 22). GH is produced and secreted not only by the pituitary but also locally by the gonads, uterus, placenta and mammary glands (7, 23). Different from the sexually dimorphic pulsatile nature of the pituitary secretion of GH, the GH secreted outside the pituitary is produced more continuously at lower concentrations (24, 25). GHRs are expressed in ovarian granulosa, theca cells, oocytes, cumulus cells, mammary glands, placenta and uterus (23). Binding of GH with its receptors can activate the JAK-STAT (Janus kinase-signal transducer and activator of transcription) pathway (26) to adjust steroidogenesis and gametogenesis, promote proliferation, development and maturation of the gonadal cells and follicles, and regulate secretion and response of gonadotropins besides improving endometrial receptivity and embryos quality.

### Signaling Pathway of GH Action

**Figure 1:**
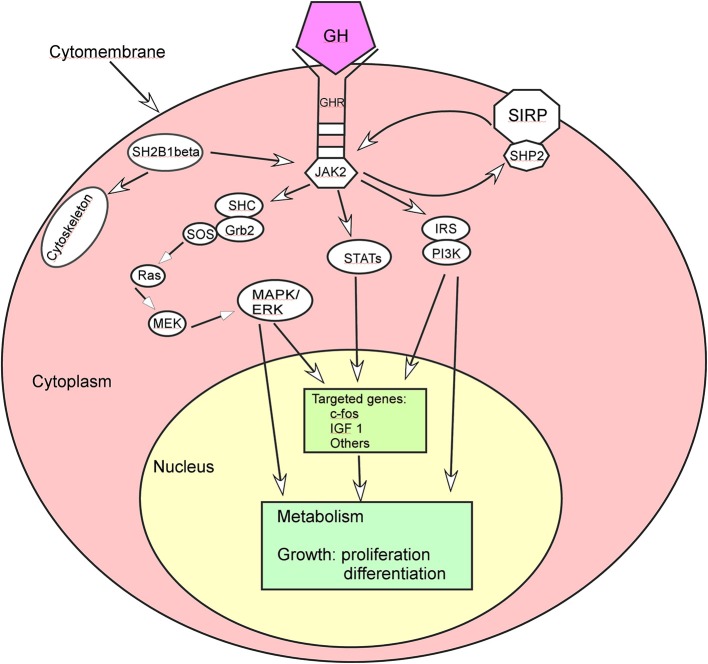
Growth hormone (GH) acts through some signal pathways. ERK, extracellular signal-regulated kinase; GHR, growth hormone receptor; Grb, growth factor receptor-bound protein; IRS, insulin receptor substrate; IGF 1, insulin-like growth factor 1; JAK2, Janus kinase 2; MAPK, mitogen-activated protein kinase; MEK, dual specificity mitogen-activated protein kinase 2; PI3K, phosphatidyl inositol 3 kinase; SHC, SH2-domain containing transforming protein; SIRP, signal regulatory protein; SOS, son of sevenless; STAT, signal transducer and activator of transcription.

GH exerts its diverse effects on body growth and metabolism by binding to its membrane-bound receptors (Figure 1). The binding of GH to its receptors increases binding of JAK2 to the GHR, activates JAK2 and stimulates tyrosyl phosphorylation of both JAK2 and GHR (27). Activation of JAK2 is a critical initial step and activates multiple signaling pathways and cellular responses for GH effects, including 1) STAT transcription factors in the expression of multiple genes like the gene encrypting IGF-1, 2) SHC adaper proteins which activate the Grb2-SOS-Ras-Raf-MEK-ERK 1,2 pathway, 3) insulin receptor substrate (IRS) proteins in the phosphatidylinositol-3-kinase (PI3K) and Akt pathway, 4) signal regulatory protein α (SIRPα), a transmembrane scaffold protein which recruits proteins like the tyrosine phosphatase SHP2, and 5) SH2B1 which is a scaffold protein activating JAK2 and enhancing GH regulation of the actin cytoskeleton.

In the STAT transcription factors implicated in the expression of multiple genes, GH binding to GHR activates JAK2 which in turn phosphorylates GHR on multiple tyrosines to subsequently recruit various STAT proteins. These STAT proteins are phosphorylated in turn by JAK2 on a critical tyrosine. After phosphorylation, the STAT proteins are released from the GHR/JAK2 complex before dimerization and move to the nucleus to bind to the STAT binding sites in GH-regulated genes, affecting metabolism, growth and development of cells.

GH binding to its receptor can also activate the MAPK (mitogen-activated protein kinase) pathway by promoting binding of the Src homology 2 (SH2) domain of SHC adapter protein to JAK2-GHR complexes, tyrosyl phosphorylation of the three forms of SHC, and binding of the adapter protein Grb2 to SHC, regulating the target genes and subsequent cell growth and differentiation (28, 29). GH can promote association of the guanine nucleotide exchange factor SOS with SHC and activate Ras, Raf, MEK and Erks (extracellular signal-regulated kinase) via a SHC-Grb2-SOS-Ras-Raf-MEK-Erk1/2 pathway. Erks adjust some different kinds of molecules like protein kinases, phospholipases, cytoskeletal proteins, and transcription factors, thus exerting multiple effects in GH targeted cells (30).

Another pathway that GH regulates is the IRS-PI3K (insulin receptor substrate-phosphatidylinositol-3-kinase) pathway: GH stimulates tyrosyll phosphorylation of both IRS 1 and 2, binding of the p85 regulatory subunit of PI3K to IRS 1 and 2 and tyrosine phosphatase SHP 2 to IRS 2, thus regulating glucose transport and other cellular responses (31–33). Activation of the IRS proteins by GH also indicates a pathway through which GH activates the transcription factor C/EBPβ. Activation of PI3K can convert phosphatidylinositol (3,4)-bisphosphate (PIP2) lipids to phosphatidylinositol (3,4,5)-trisphosphate (PIP3) to recruit Akt to the plasma membrane for the kinase PDK1 to access and phosphorylate T308 in Akt, resulting in partial activation of Akt (34). Akt phosphorylation of glycogen synthase kinase 3 (GSK3) leads to inhibition of GSK3 activity which decreases phosphorylation of GSK3 phosphorylation site in C/EBPβ, and elevated binding of a form of C/EBP designates liver activating protein (LAP) to the c-fos promoter for regulating cell proliferation and differentiation (35). The maximal expression of c-fos needs input from multiple GH signaling pathways, and the promoter region of c-fos includes a binding site for STAT 1 and 3 hetero or homodimers whose binding enhances c-fos gene expression (36–38).

SIRPα is a transmembrane glycoprotein recruits multiple SHP2 proteins, and activation of JAK2 by GH binding to GHR can highly phosphorylates SIRPα1 and recruits SHP 2 tyrosine phosphatases for negative regulation of GH-JAK2 signaling. SH2B1 is a scaffold protein to activate JAK2 and enhance GH regulation of the actin cytoskeleton.

### Effect of GH on Ovarian Reactivity

As one of the targets of GH action, the ovary can be directly regulated by GH for its reactivity to gonadotropins. At the same time, GH can indirectly influence the ovarian function through IGF-I. Ovarian granulosa cells produce IGFs and express IGF receptors, and the IGFs and the receptors form a paracrine/autocrine system together with IGF binding proteins (6). Binding of IGF-I with its receptor can activate the phosphatidylinositol 3-kinase (PI3K)/protein kinase B (Akt) signaling pathway to stimulate and regulate normal follicular growth and development in synergy with gonadotropins to increase the luteinizing hormone receptor level, consequently raising the ovary sensitivity to the follicule-stimulating hormone (FSH) (5).

### Effect of GH on Follicle Development

GH is a potent activator of proliferation and differentiation of the ovarian follicles, and its administration generally increases ovarian weight and follicular size and number but inhibits follicular atresia (6, 39). GH is necessary for optimal follicular maturation and survival because GH addition to *in vitro* maturation medium of primordial and immature follicles can promote activation, survival and development of preantral follicles originating from sheep, goats and mice (40–42). Besides enhancing proliferation of the thecal and granulosa cells in the immature preantral follicles of mice (43), GH can improve the oocyte retrieval and fertilization rate in human oocytes subjected to *in vitro* maturation (44) and promote cumulus expansion and subsequent embryo development in rhesus macaque (45). *In vivo* GH can also improve the number of developing follicles in mice, buffalo and sheep (46–48), promote proliferation and inhibit apoptosis in the ovarian stroma and small follicles in chickens (49) as well as increase the follicle size in undernourished cows (50). Expression of GH in transgenic mice and adult sheep can promote follicular development besides increasing the ovary weight, ovulation rate, and the size and health of ovarian follicles (51, 52). In goat antral follicles, GH can indirectly adjust the early development stage but control the late stage formation of follicles through the GHR (53), and supplementation of GH to *in vitro* culture of caprine preantral ovarian follicles can increase the antrum formation rate, percentage of oocytes resuming meiosis and mature oocytes (42). This is because GH can induce granulosa and thecal cells to produce IGF-I, which can regulate the ovarian function to resume meiosis of the oocytes through autocrine/paracrine function. GH can also improve the mitochondria activity to directly ameliorate the oocyte quality (54). With aging, the number of functional mitochondria will be decreased, leading to impaired separation of chromosome associated with failed fertilization. Administration of GH in older women can upregulate expression and activity of GHRs, beneficial to improving the mitochondrial function, quality of oocytes and fertilization rate (54).

### Effect of GH on Endometrial Receptivity

Good endometrial receptivity is the precondition for embryo implantation. During the treatment process of ovulation promotion with IVF, supra-physiological levels of estrogen regulate effects of endogenous hormones on endometrial thickness and pattern and expression of receptors and related factors to subsequently affect the endometrial receptivity (55). The uterus produces GH, which in turn adjusts the uterus (6), increases endometrial blood flow and expression of related cytokines and subsequently improves the endometrial receptivity (56). GH can also promote expression of endometrial vascular endothelial growth factor (VEGF)-1, leukemia necrosis factor, and matrix metalloproteinase 9, resulting in proliferation of endometrial glands, expansion of glandular cavity, blood vessel formation, and differentiation, thickening of endometria and endometrial mesenchyme (5, 57). The endometrial receptivity is consequently improved. In addition, GH can increase synthesis of IGF-I in the ovary, enable the pituitary gland to secrete more FSH and promote secreting function of the granulosa cells so as to increase the estrogen level for improving the endometrial thickness and pattern.

## Application of GH in IVF

In 1988, Homberg et al. (58) found that GH increases the ovary sensitivity to the ovulation-inducing effect of gonadotropins, with significant reduction in the amount, duration of treatment and daily effective dose of human menopausal gonadotropin caused by GH addition. In an randomized clinical trial, Owen et al. demonstrated that GH improved the ovarian response to conventional ovarian stimulation regimens in females with poor ovarian responses, with significantly (*P* < 0.05) more follicles and more oocytes obtained in patients with polycystic ovaries when GH (24 IU) was administered on alternate days concurrently with the gonadotrophin treatment after enrollment (59). Despite numerous studies, GH supplementation to the IVF regimen of poor ovarian responders remains controversial (60). Some studies suggested that pretreatment of GH could increase ovarian response to gonadotropins, improve oocyte quality and consequently be applied in the pituitary down-regulation cycle or in poor ovarian response to gonadotropins in the IVF (16, 61–64). Other authors did not support GH as an effective adjuvant for infertility treatment because the live birth rate was not increased even though some benefits might have been achieved through the use of GH (62, 65–67). However, some authors performed large-scale meta-analyses which supported GH as a useful *in vivo* adjuvant for human protocols (9, 10, 68). After analyzing six randomized controlled trials in a meta-analysis, Kolibianakis et al. found that GH addition significantly increased the clinical pregnancy rate by 16% (95% CI 4–28), the live birth rate by 17% (95% CI 5–30) and the proportion of patients reaching embryo transfer by 22% (95% CI 7–36) in poor-responding patients undergoing ovarian stimulation for IVF (10). Duffy et al. also performed a meta-analysis including ten randomized controlled trials to evaluate effectiveness of adjuvant GH in poor responders in IVF (9), and they found that a statistically significant difference in both the live birth rate (OR 5.39, 95% CI 1.89–15.35) and pregnancy rate (OR 3.28, 95% CI 1.74–6.20) favoring the use of adjuvant GH in IVF protocols for women considered poor responders without increasing adverse events. In another systematic review and meta-analysis including 22 eligible randomized controlled trials assessing interventions to improve the pregnancy rate in poor responders undergoing IVF (68), it was found that the only interventions that appear to increase the probability of pregnancy were the addition of GH to ovarian stimulation (OR for live birth: 5.22, 95% CI 1.09–24.99) and the performance of embryo transfer on day 2 compared with day 3 (the pregnancy rate was increased by 11.4%, 95% CI: 1.6–21.0). However, some randomized controlled trials discouraged the use of GH in IVF because no definitive benefits have been demonstrated in increasing the live birth rate for poor responders (18, 19), but careful evaluation of these trials showed severe drawbacks as stated before. Up to now, GH has been widely applied in the reproduction area but primarily for poor ovarian responders (7, 8, 10, 11, 14, 16, 19, 60–63, 67–76), poor quality of embryos (16, 17, 67, 70, 74, 77), improper endometrial reaction (5, 12, 56, 78, 79) and repeated failure of embryo transfer (1, 63, 80–82).

### GH Application for Improving Ovarian Response

GH application combined with gonadotropins for ovulation promotion can improve the pregnancy outcome in most patients with poor ovarian responses. Lattes et al. (16) performed a prospective controlled study in 64 poor responders to previous IVF cycles who failed to achieve clinical pregnancy and were supplemented with low-dose GH in a subsequent cycle with the same gonadotropin dose and protocol. It was found that daily administration of low-dose (0.5 IU) GH from the first day of the GnRH agonist until the day of hCG application could significantly increase the clinical pregnancy rate (34.4 vs. 0%), number of both top quality embryos (1.03 ± 1.17 vs. 0.64 ± 0.88, *P* = 0.046) and cryopreserved embryos (0.85 ± 1.49 vs. 0.30 ± 0.81, *P* = 0.02). In a prospective controlled trial investigating the efficacy of GH pretreatment within an antagonist protocol in IVF/ICSI (intracytoplasmic sperm injection) cycles in poor responders, use of low-dose (4IU/d) GH on the start of ovarian stimulation could significantly decrease the effective dose of gonadotropins (median 750, low quantile (LQ) 533.3 and upper quantile (UQ) 1312.5 for GH group vs. 1375, 862.5, and 2962.5 for non-GH group, respectively) and duration of stimulation (median 8d, LQ 7d and UQ 10 d for GH group vs. 9d, 8d, and 10d for non-GH group, respectively), but increase the total number of oocytes (median 4, LQ 3 and UQ 7 for GH group vs. 3, 2 and 4 for non-GH group, respectively), metaphase II stage oocytes (median 2, LQ 1 and UQ 6 for GH group vs. 1, 0 and 2 for non-GH group, respectively), two pronucleus zygotes (median 2, LQ 0 and UQ 3 for GH group vs. 1, 1 and 2 for non-GH group) and good-quality transferred embryos (median 1.5, LQ 1 and UQ 2 for GH group vs. 0, 0 and 1 for non-GH group, respectively), with ultimate increase in the clinical pregnancy rate (21.74 vs. 0%) (74). A meta-analysis studying the influence of different GH addition protocols to poor ovarian responders on clinical outcomes in controlled ovary stimulation cycles demonstrated that either high- (12IU/d or 24 IU/qod) or low-dose (2IU/qod) GH could significantly improve the clinical pregnancy (RR 1.76, 1.25–2.48) and the live birth rate (RR 1.91, 1.29–2.83) in poor ovarian responders even though GH supplementation in the middle of luteal phase did not increase the pregnancy and live birth rates (11). In a prospective randomized clinical trial investigating GH as an adjuvant therapy added to either long or short agonist protocol, miniflare or antagonist protocols with GH introduced on day 6 of human menopausal gonadotropin stimulation in a dose of 2.5 mg S.C. daily till the day of hCG administration, the long GH agonist protocol was superior to the other three protocols regarding the number of oocytes retrieved (5.06 ± 1.83 vs. 4 ± 1.69, 4.95 ± 1.9 and 4.98 ± 2.15) and fertilized (3.73 ± 1.47 vs. 3.02 ± 1.51, 2.89 ± 1.14 and 3.57 ± 1.41) (83). But the clinical pregnancy rate was not significantly different among the four different protocols (36.7 vs. 23.2, 25.9, and 30.4%, *P* > 0.05) even though there was a difference in favor of the long GH agonist. Since the long GH agonist protocol required significantly greater gonadotropin dose and longer duration of stimulation, low-dose GH was suggested for GH supplementation protocol because low-dose GH could improve the reactivity of the ovary.

### GH Application for Improving Oocyte Quality

In a sequential crossover study of IVF to evaluate GH supplementation in poor-prognosis patients based on the past failure to conceive due to low response to high-dose stimulation (<3 metaphase II oocytes) or poor-quality embryos (17), GH supplementation (10 IU) could significantly improve the clinical pregnancy rate (20 vs. 9%, *P* < 0.05) per fresh transfer and per frozen-thawed embryo derived from GH cycles leading to a highly significant productivity rate (30 vs. 14%, *P* < 0.001). These GH effects were significant across all age groups, especially in younger patients (24 vs. 10% for patients <35 years but 15 vs. 11% for >40 years), and independent of stimulation modality or number of transfers. In this study (17), GH (10 IU) was injected in the previous cycle on days 7, 14 and 21 with a final injection on day 2 of the treatment cycle for the first 4 years of the study, and for the last 2 years of the study, patients received six injections with the first beginning on day 21 of the preceding cycle and the subsequent injections being on days 2, 6, 8, 10 and 12 at the dose of 10 IU. Nonetheless, it was suggested that longer pretreatment (4–6 weeks before gonadotropin start) with low physiological dose of 2 IU/d GH might be more beneficial to follicular growth and development. GH is beneficial to the repair of oocytes and quality improvement of ova in older patients because it can upregulate expression of IGF-I in the ovary and stimulate production of oocyte-derived growth and differentiation factor and bone morphogenetic protein-15 (84). After studying the outcomes of poor responders following GH pretreatment (4.5 IU GH administered once every 2 days since day 16 of the previous cycle for six times and once every 2 days since stimulation day 1 for three times) with IVF/ICSI mild stimulation protocol (100 mg Clomiphene citrate administered daily from day two or three of menstrual cycle) in a retrospective analysis of 132 patients whose data were prospectively enrolled and maintained, Chu et al found that GH supplementation could significantly increase the good-quality embryo rate in either IVF (68.1 vs. 51.5%, *P* = 0.008) or ICSI (53.9 vs. 36.7%, *P* = 0.045) group (61). In an observational study investigating GH adjuvant therapy in patients with three or more IVF failures (15), GH supplementation in the dose of 8 IU administered from day 1 of stimulation until the trigger day could significantly increase the pregnancy rate (25.7 vs. 18.2%, *P* < 0.01) per retrieval in these patients, with the pregnancy rate being elevated to a level similar to that observed in the study center for the whole population. An improvement of cytoplasmic competence is proposed as an explanation for this.

### GH Use for Improving the Uterine Receptivity

Adequate thickness of the endometrium is the key to successful implantation, and a thin endometrium is critical in implantation failure (85–87). A thin endometrium may be caused by impaired endometrial growth which is closely related to angiogenesis and uterine blood flow. Angiogenesis is necessary for endometrial growth following menstruation and can provide a vascularized receptive endometrium for implantation (88, 89). Uterine blood flow is also an important factor for endometrial growth and is closely related to endometrial vascular development (86, 90, 91). Low uterine blood flow may cause a decreased pregnancy rate in patients with IVF-ET (embryo transfer), suggesting a close relationship of uterine blood flow with uterine receptivity (92, 93). High blood flow impedance of the uterine and radial arteries, poor growth of glandular epithelium, decreased VEGF and poor vascular development have all been confirmed to be characteristic of a thin endometrium (86). High blood flow impedance in the uterine and radial arteries may impair glandular epithelium growth and decrease endometrial VEGF level, and low VEGF level may result in poor vascular development, further decreasing the endometrial blood flow. This is a vicious circle which may lead to a thin endometrium associated with poor endometrial receptivity. In a randomized controlled trial investigating effects of GH on uterine receptivity in women with repeated implantation failure in an oocyte donation program, it was demonstrated that administration of GH (dose and timing were not mentioned in the study) could significantly (*P* < 0.05) increase the endometrial thickness (9.3 ± 1.5 vs. 8.6 ± 1.0 mm), implantation rate), pregnancy (54.3 vs. 17.1% with the OR of 6.9 and 95% CI 2.2–22.5) and live birth rates (51.4 vs. 17.1%, with the OR 6.4 and 95% CI of 2.0–20.9), with no abnormality detected in any of the babies born (1). Consistent with the above results, the randomized controlled trial by Bassiouny et al also proved the protective effects of GH on the endometrial thickness during IVF (8), and in this trial with 141 patients randomized into two groups for GH or not, GH was administered on day 6 of human menopausal gonadotropin stimulation in a daily dose of 2.5 mg SC (equivalent to 7.5 IU) until the day of hCG triggering and significantly (*P* < 0.05) increased the endometrial thickness (12.14 ± 1.25 mm vs. 11.56 ± 1.56 mm) besides significant increase in number of collected oocytes (7.58 ± 1.40 vs. 4.90 ± 1.78), number of MII oocytes (4.53 ± 1.29 vs. 2.53 ± 1.18), number of fertilized oocytes (4.04 ± 0.96 vs. 2.42 ± 1.03) and number of transferred embruyos (2.89 ± 0.45 vs. 2.03 ± 0.82). After investigating the effects of GH on clinical outcomes following frozen-thawed embryo transfer in a prospective controlled study with 4 IU GH injected subcutaneously daily from day three of the menstrual cycle until the day of progesterone injection, Wang et al. (56) found that addition of GH could significantly (*P* < 0.05) increase the clinical pregnancy (49.4 vs. 32.5%), embryo implantation (22.7 vs. 14.3%) and live birth (41.6 vs. 24.7%) rate. The serum levels of estradiol (798.73 ± 654.13 vs. 602.32 ± 438.9 pmol/L) and IGF-1 (25.55 ± 2.87 vs. 24.37 ± 3.06 nmol/L), endometrial thickness (9.6 ± 1.0 vs. 9.2 ± 0.8 mm) and serum level of VEGF (251.03 ± 39.48 vs. 227.93 ± 36.94 ng/L) were also significantly (*P* < 0.05) increased by addition of GH (56). All these effects may be caused by GH to increase the endometrial blood perfusion and expression of cytokines related to endometrial receptivity. After investigating effects of GH on pregnancy rate of patients with thin endometrium in a randomized controlled study with GH administered in a daily subcutaneous injection dose of 5 IU on day 3 of their cycles until the 18th day, Cui et al. (5) found that GH treatment could significantly (*P* < 0.05) increase the endometrium thickness on day 3 (7.87 ± 0.72 vs. 6.34 ± 0.86), the implantation rate (24.4 vs. 10.5%) and clinical pregnancy rate (42.5 vs. 18.9%) compared with the control group. Moreover, administration of GH significantly up-regulated expression of VEGF, integrin beta 3 and IGF-I at both mRNA and protein levels (5). The integrin beta 3 is a generally accepted biomarker of uterine receptivity (94) and is decreased in patients with unexplained infertility, endometriosis, luteal deficiency and lower pregnancy rates (95, 96). Increase of integrin beta 3 in GH-treated patients provides evidence that GH has a positive effect on the improvement of endometrial receptivity and pregnancy outcomes (1, 5).

## Administration Protocol of GH

Currently, the addition protocols of GH for poor ovarian responders in IVF include addition at 4–6 weeks before hCG administration, in the luteal phase of the preceding menstrual cycle (in the pituitary down-regulation phase within a gonadotropin-releasing hormone agonist long protocol), at the time of hCG administration, and at the middle and late follicular phases. The dose for GH is from 0.5IU/d to 12 IU/d, but a small dose was preferred in a recent study (3-6 IU/week) (7).

### 4–6 Weeks Before hCG Administration

Because this is at the antral follicle stage, the GH protocol should be of small doses with a long course of treatment. In a single-center retrospective study of GH supplementation in IVF patients classified as poor prognosis, Kevin et al. (77) treated these patients with GH administration on days 2–3 during the preceding menstrual cycle by hypodermic injection in the dose of 1.5 IU/d for 6 weeks prior to trigger with hCG. It was demonstrated that GH could significantly increase the implantation, clinical pregnancy by 3.42 fold (95% CI 1.82 to 6.44, *p* < 0.0005) and live birth rates by 6.16 fold (95% CI 2.83 to 13.39, *p* < 0.0005) despite these patients being significantly older with lower ovarian reserve than the control group. Cui et al. (5) also treated patients with thin endometrium by injecting GH (5 IU) subcutaneously on day 3 of their cycles until the 18th day, and the endometrium thickness (7.87 ± 0.72 vs. 6.34 ± 0.86), implantation (24.4 vs. 10.5%) and clinical pregnancy (42.5 vs. 18.9%) rates were all significantly increased compared with the control group.

### At the Luteal Phase of the Preceding Menstrual Cycle

GH is mostly administered in the pituitary down-regulation phase for a gonadotropin-releasing hormone agonist long protocol at this stage. In a randomized prospective clinical trial investigating GH co-treatment within a gonadotropin-releasing hormone agonist long protocol in patients with poor ovarian response (14), poor responding women achieved more oocytes (6.5 ± 2.1 vs. 3.2 ± 1.4, *P* < 0.001) and higher fertilization rates (4.4 ± 1.8 vs. 1.5 ± 0.9) with decreased doses and duration of gonadotropin when GH was added in the pituitary down-regulation in the dose of 12 IU/d until the day of hCG. However, use of low-dose GH of 0.5IU/d until the hCG day in the luteal phase for pituitary down-regulation in a prospective self-controlled study could achieve a greater number of top quality embryos (1.03 ± 1.17 vs. 0.64 ± 0.88) and cryopreserved embryos (0.85 ± 1.49 vs. 0.3 ± 0.81) as well as an increased clinical pregnancy rate (34.4% vs. 0) (16). Nonetheless, some other studies had also pointed out that GH supplementation in the middle luteal phase did not increase the clinical pregnancy and live birth rates (14, 62, 72, 73). One retrospective case-control study which used GH supplementation in the middle luteal phase in a daily dose of 3.33 mg of GH administered in subcutaneous injection (62) reported no improvement in IVF cycle outcomes with the similar clinical pregnancy rate (29 vs. 32 %, *P* = 0.99), mean number of mature oocytes retrieved (2.5 vs. 5.0, *P* = 0.13) and mean number of embryos available for cryopreservation (0 vs. 0). In one randomized prospective study by Kucuk et al. (14) which used GH in the daily subcutaneous injection dose of 4 mg (equivalent to 12 IU) from day 21 of preceding cycle along with GnRHa until the day of hCG, although more pregnancies (38.7 vs. 20%) and more clinical pregnancies (32.3 vs. 16.7%) with fetal heart activity have been achieved in the GH group compared with the control group, the difference did not reach the statistical significance (*P* < 0.05). In the randomized prospective study on 82 poor responders (72), patients in the GH group received daily injection of 4 IU GH from day 21 of previous cycle until the day of hCG injection, and the reproductive outcomes were similar (*P* > 0.05) in both groups with similar chemical pregnancy (15 vs. 14. 3%) and clinical pregnancy rate (12.5 vs. 11.2%).

### At the Start of Gonadotropin

One study performed in China demonstrated that supplementation of GH in the dose of 4.5IU/d at the start of gonadotropin could significantly increase the number of oocytes collected, metaphase II and fertilized oocytes, and top quality embryos (97). Although the biochemical pregnancy rate (36.45%), clinical pregnancy rate (22.51%), implantation rate (18.32%) and live birth rate (19.1%) were all higher in the GH than the control group (32.63, 20.98, 18.18, and 15.85%, respectively), no significant difference (*P* > 0.05) existed. This study had 90 women in either the GH or the control group and was performed in a 2-year period, and enrollment of more patients with increased period of study may probably increase the statistical power with the significance level surpassing 0.05.

### At the Middle and Late Follicular Phases

In two randomized prospective clinical trials investigating the effects of GH added to the antagonist protocol in the IVF/ICSI cycles for patients with poor ovarian responses, when the GH was added on the 6th day in the dose of 2.5 mg/d (equivalent to 7.5 IU/d) until the hCG day, the mean number of oocytes collected, metaphase II and fertilized oocytes, top quality oocytes and endometrial thickness were all significantly increased but the clinical pregnancy rate was not significantly improved (11, 62). When GH was given in a daily subcutaneous dose of 8 IU from day 7 of exogenous gonadotrophin administration till the day following hCG triggering in women older than 40 years (13), patients in the GH –treatment group received slightly more embryos per transfer compared to the placebo group (4.2 vs. 3.5, *P* > 0.05), but significantly (*P* < 0.05) higher clinical pregnancy rate (26 vs. 6%) and clinical implantation rate (6.2 vs. 1.7%). The delivery rate (22 vs. 4%) and live birth (5.2 vs. 1.1%) rate were also significantly higher in patients with GH supplementation compared with controls.

## Subjects and Benefits of GH Treatment

As stated before, GH adjuvant therapy was clinically widely used in poor ovarian responders (7, 8, 10, 11, 14, 16, 19, 60–63, 67–76), poor quality of embryos (16, 17, 67, 70, 74, 77), improper endometrial reaction (5, 12, 56, 78, 79) and repeated implantation failure (1, 63, 80–82). When it is used in patients with repeat implantation failure which is defined as failure of pregnancy despite implantation of a high-quality embryo at least three times or of over 10 embryos on repeat implantation failure (1, 63, 80–82), the mechanism of this action is, as stated before, related to GH stimulating proliferation and differentiation of granulosa cells, increasing production of estradiol in both early and late follicular development for animal and human ovaries, enhancing effect of FSH on the development of ovarian follicles and improving endometrial thickness (82, 98–100). A recent randomized controlled clinical trial performed in China of GH co-treatment on controlled ovarian stimulation in normal ovarian response women showed significantly (*P* < 0.05) higher two pronuclei rate (33.92 vs. 30.92%) and higher quality embryo rate (63.4 vs. 59.33%) besides significantly increased number of embryos available (3.79 ± 2.74 vs. 2.90 ± 2.12, *P* < 0.001) and higher endometrial thickness on hCG day (11.96 ± 2.24 vs. 11.62 ± 2.81, *P* = 0.036) in 781 patients receiving GH of 1IU/4IU administered daily since day two of the previous cycle or day two in accordance with controlled ovarian stimulation until hCG trigger in comparison with the control group without GH adjuvant therapy (79). Among a total of seven systematic reviews and meta analyses found online (pubmed) up to 2019 investigating the effect of GH adjuvant therapy on poor responders undergoing IVF (9–11, 65, 67, 68, 101), only two meta analyses demonstrated no improvement in the liver birth rate (65) or the clinical pregnancy rate (OR 0.051, 95% CI −0.033 to 0.134, *P* = 0.197) (67). All the other five meta analyses showed that GH supplementation in IVF significantly increased the live birth rate with the OR 5.22 and 95% CI 1.09–24.99 in the study by Kyrou et al. reported in March 2009 (68), OR 6 and 95% CI 3–20 in the study by Kolibianakis et al presented in the November-December issue of 2009 (10), OR 5.39 and 95% CI 1.89–15.35 in the study by Duffy et al published in January 2010 (9), OR 2.96 and 95% CI 1.17–7.52 in the study by Jeve et al. published in the April-June issue of 2016 (101), and OR 1.91 and 95% CI 1.29–2.83 in the latest study by Li et al. presented in March 2017 (11). Although some individual randomized controlled studies did not reveal significant improvement in the live birth rate in GH supplementation in women undergoing IVF, these pooled data in most meta-analyses favored the use of GH for IVF because of the significantly increased live birth rate.

In conclusion, GH supplementation in the process of IVF might improve reactivity of ovary, endometrial receptivity, clinical pregnancy and live birth rates. Although GH is frequently used as an adjuvant in patients with poor ovarian response for ovulation promotion and in patients with repeated implantation failure for improving the endometrial receptivity, no clear standards have currently been set up for the indications, methods and dosages in clinical application, and more in-depth studies are consequently needed for appropriately addressing these issues.

## Author Contributions

Y-MX, G-MH, and B-LG contributed substantially to the following aspects:

1. Substantial contributions to the conception or design of the work, or the acquisition, analysis or interpretation of data for the work.
2. Drafting the work or revising it critically for important intellectual content.
3. Provide approval for publication of the content: G-MH and B-LG.
4. Agree to be accountable for all aspects of the work in ensuring that questions related to the accuracy or integrity of any part of the work are appropriately investigated and resolved.

### Conflict of Interest Statement

The authors declare that the research was conducted in the absence of any commercial or financial relationships that could be construed as a potential conflict of interest.
